# Global burden-integrated analysis of osteoarthritis research-to-translation dynamics, 1999–2023

**DOI:** 10.1371/journal.pone.0349128

**Published:** 2026-05-26

**Authors:** Riya Mukherjee, Chung-Ming Chang

**Affiliations:** 1 Graduate Institute of Biomedical Sciences, Chang Gung University, Taoyuan, Taiwan; 2 Master & PhD Program in Biotechnology Industry, Chang Gung University, Taoyuan, Taiwan; The Affiliated Changzhou No 2 People's Hospital of Nanjing Medical University, CHINA

## Abstract

**Background:**

Osteoarthritis (OA) is a leading cause of global disability, yet the temporal relationship between disease burden and research and translational activity has not been systematically examined using an integrated, system-level framework.

**Methods:**

We conducted a retrospective, system-level analysis of osteoarthritis (OA) research and disease burden from 1999 to 2023. Annual global OA burden estimates (prevalence, YLDs, and DALYs) were obtained from the Global Burden of Disease framework and harmonized with year-wise data on OA publications, experimental model use, interventional clinical trials, and funding acknowledgements derived from PubMed and ClinicalTrials.gov. Publication domains were classified using predefined, rule-based queries applied to metadata fields. Temporal trends, proportional composition, and burden–research scaling relationships were evaluated using descriptive regression and proportion-based analyses.

**Results:**

Between 1999 and 2023, global osteoarthritis (OA) disability-adjusted life years (DALYs) approximately doubled, while annual OA publication volume increased more than fourfold. Log–log regression indicated elastic scaling of research output relative to DALYs (β > 1), reflecting disproportionate growth in publication activity. In contrast, interventional clinical trial initiation peaked in the late 2000s and declined in recent years, with trial activity representing a consistently small fraction of total OA publications. Human-relevant experimental approaches constituted a modest and relatively stable proportion of OA research over time. Lag-association analyses demonstrated strong temporal co-trending between DALYs and total publications, moderate alignment for human-relevant research, and weaker associations for clinical trial activity.

**Conclusion:**

Over 1999–2023, osteoarthritis research activity expanded substantially relative to disease burden, while downstream clinical translation and adoption of human-relevant approaches remained comparatively limited.

## Introduction

Osteoarthritis (OA) is among the leading causes of chronic disability worldwide, contributing substantially to pain, functional limitation, and long-term loss of productivity. Global epidemiologic assessments consistently demonstrate a high and rising OA burden, underscoring its substantial public health impact and socioeconomic consequences [1,2]. Despite this extensive health and socioeconomic impact, OA remains characterized by a persistent absence of disease-modifying therapies, with current clinical management largely focused on symptomatic relief rather than structural disease alteration [3]. This contrast between population-level disease burden and limited disease-modifying therapeutic options has positioned OA as a longstanding challenge in musculoskeletal medicine.

Previous investigations have examined individual components of the osteoarthritis research and translational landscape, including epidemiologic burden, clinical trial activity, research funding, and scientific publication output. However, these domains are typically analyzed in isolation, limiting the ability to evaluate how research activity and translational efforts evolve relative to population health need at a systems level [4–6]. As a result, it remains difficult to determine whether observed translational challenges in OA primarily reflect biological complexity or broader structural patterns across research, funding, and methodological domains [2,4,7]

Within this context, burden-anchored evaluation offers a descriptive perspective rather than a causal or prescriptive one. Disability-adjusted life years (DALYs) provide a standardized measure of population-level health loss and serve as a common reference scale against which diverse research and translational indicators can be contextualized [8]. Importantly, burden anchoring does not assume that research investment or clinical trial activity should scale linearly or deterministically with DALYs. Instead, it enables comparison of temporal scaling patterns across domains, highlighting concordance or divergence without implying causality or normative performance thresholds [4,6].

In this study, we implement a multidimensional, descriptive analytical approach to examine long-term temporal trends across key stages of the osteoarthritis research-to-translation pathway, with epidemiologic burden used strictly as contextual reference rather than as a mechanistic driver. Specifically, we analyze system-level indicators spanning scientific publication output, interventional clinical trial initiation, adoption of human-relevant and experimental research methodologies (including in vitro, ex vivo, and microphysiological systems), and funding acknowledgement coverage derived from publication metadata [9,10]. Public funding is interpreted as an upstream enabling context rather than as a direct measure of translational success.

Using publicly available datasets spanning 1999–2023, this study provides a structured characterization of how key components of the osteoarthritis research landscape have evolved over time relative to global disease burden. By integrating epidemiologic, bibliometric, clinical trial, methodological, and funding acknowledgement indicators within a consistent temporal framework, the analysis aims to clarify patterns of temporal concordance and divergence across domains while acknowledging the constraints inherent to aggregate, secondary data sources. This burden-anchored perspective is intended to support transparent assessment of translational dynamics in osteoarthritis while acknowledging the constraints inherent to aggregate, secondary data sources [4,11].

The primary objective of this study is to characterize long-term temporal trends in osteoarthritis research activity in relation to global disease burden from 1999 to 2023. Specifically, we aim to (i) describe changes in overall scientific publication output, (ii) evaluate patterns in interventional clinical trial initiation, (iii) assess shifts in experimental model adoption, including human-relevant methodologies, and (iv) examine funding acknowledgement coverage within OA publications. By anchoring these indicators to epidemiologic burden as a contextual reference, the study seeks to provide a structured assessment of research burden dynamics over time (Table 1).

**Table 1:** Study Summary: Osteoarthritis Research–Burden Ecosystem (1999–2023).

Section	Summary
Background	Osteoarthritis (OA) is a leading cause of global disability, yet the extent to which research activity has aligned with disease burden and progressed effectively across translational stages remains unclear. This study adopts a longitudinal systems-level framework (1999–2023) to evaluate research–burden scaling, translational composition, funding acknowledgement coverage, and temporal responsiveness within the OA research ecosystem.
Main findings and limitations	OA publication output expanded substantially over the study period and increased more rapidly than global OA DALYs, indicating amplified growth relative to disease burden. However, translational distribution remained uneven, with preclinical animal-model research constituting a dominant share of output, while human-relevant approaches increased more gradually and clinical trial activity demonstrated comparatively modest expansion. Funding acknowledgement coverage captured in PubMed metadata rose more than tenfold, with NIH institutes most frequently acknowledged, although these findings reflect structured metadata and do not represent funding magnitude or investment levels. Lagged response analyses demonstrated strong short-term co-trending between aggregate publication volume and burden, but weaker concordance in downstream clinical activity. All findings are based on bibliometric and metadata-derived indicators and do not infer causality or research quality.
Policy implications	The observed divergence between rapid publication expansion and comparatively slower translational progression underscores the need for differentiated research policy strategies. Strengthening support for human-relevant and integrative translational models may enhance efficiency from preclinical discovery to clinical evaluation. Systematic monitoring of research–burden alignment using real-time indicators could inform adaptive funding prioritization. Improved standardization and transparency of funding metadata would further strengthen future assessments of research investment patterns in OA and other chronic non-communicable diseases.

## Methods

### Study design

This study was designed as a retrospective, system-level analysis examining how osteoarthritis (OA) research activity has evolved in relation to global disease burden between 1999 and 2023. Using publicly accessible databases, we compiled annual data on OA publications, experimental model use, interventional clinical trials, funding acknowledgements, and global burden metrics. These datasets were harmonized by calendar year to enable direct temporal comparison. The objective was to describe long-term patterns in research growth, translational stage distribution, human-relevant model adoption, and their alignment with changes in OA burden over time. All analyses were descriptive and focused on identifying temporal trends rather than inferring causality.

### Data selection criteria and time frame

#### OA research volume.

To quantify long-term trends in osteoarthritis (OA) research activity, we conducted a retrospective, system-level analysis of OA-related publications indexed in PubMed between 1999 and 2023 (n = 25). Publications were retrieved using a comprehensive OA-focused search strategy based on controlled vocabulary and keyword terms, designed to capture the full breadth of OA research across anatomical sites and experimental contexts (S1 Table). The complete PubMed search strategy, including Boolean syntax, MeSH terms, date of search execution, and any applied filters are provided in the Supplementary Materials.

Annual OA research volume was operationalized as the number of PubMed-indexed OA-related publications per calendar year. The extracted dataset was curated to ensure consistent year coverage across the study period, and annual publication counts were compiled for descriptive analysis. Temporal trends in OA research volume were evaluated using two complementary regression approaches.

First, a linear regression model was fitted to estimate the average absolute change in publication counts per year. Second, a log-linear regression model was applied to characterize proportional growth in research output over time, allowing estimation of the average annual growth rate. For years with zero counts (if present), a small offset was applied to ensure numerical stability during log transformation. Model estimates are reported with corresponding 95% confidence intervals. All analyses were descriptive in nature and were not intended to infer causality or research prioritization. Therefore, annual counts, regression outputs, and visualization form the basis for contextualizing subsequent analyses of research composition, translational activity, and responsiveness.

#### OA disease burden.

We conducted a retrospective, descriptive time-trend analysis of osteoarthritis (OA) burden and related metrics from 1999 to 2023. Global OA burden estimates were obtained from the Global Burden of Disease (GBD) framework (subtype-aggregated OA), extracting annual prevalence, years lived with disability (YLDs), and disability-adjusted life years (DALYs) together with the GBD lower and upper uncertainty bounds for each metric. Given negligible OA mortality at the global level, DALYs were expected to be largely disability-driven and closely track YLDs.

Annual burden values were harmonized to one record per year for each metric. For each outcome, the primary estimate was used for time-trend modeling, and the uncertainty bounds were retained for visualization as shaded ribbons.

Temporal patterns were characterized using (i) linear regression of burden on calendar year to summarize absolute change over time, and (ii) log-linear regression of log(burden) on year to summarize proportional change. From log-linear models, we derived the average annual percent change as 100 X (exp (β)- 1), where β is the year coefficient. Analyses were interpreted descriptively and do not imply causality. For visualization, we overlaid a GAM smooth to highlight the overall trajectory while retaining year-specific observed points.

### Interventional clinical trial activity

Interventional osteoarthritis (OA) clinical trials were identified from the ClinicalTrials.gov registry and compiled into a yearly time series spanning 1999–2023. Records were filtered to interventional studies under the condition ‘osteoarthritis’; withdrawn trials were excluded and duplicates were handled by unique NCT identifier. Temporal activity was operationalized using the study start year as the primary time variable, and annual counts were summarized as the number of interventional OA trials per year (Supplementary Materials). Years with no recorded trials within the year window were explicitly retained as zero-count years to preserve a complete chronological series. Analyses consist of yearly aggregation and visualization of trial counts over time; a LOESS smoother was overlaid to highlight the overall temporal pattern without inferential modeling.

### Human-relevant alignment trend in osteoarthritis research

Year-wise osteoarthritis (OA) publication counts from 1999–2023 were compiled from two datasets: (i) total OA publications per year (denominator) and (ii) OA publications classified as human-relevant (numerator). Human-relevant studies were identified using a predefined, rule-based query applied to PubMed metadata, capturing publications that reported human in vitro or ex vivo systems, microphysiological models, organ-on-chip platforms, or related replacement-oriented approaches. The complete query syntax and controlled vocabulary terms are provided in the Supplementary Materials. Classification was implemented programmatically using reproducible R scripts; no record-level manual inclusion/exclusion was performed. Annual counts were merged across the full 1999–2023 window, with missing years retained as zero counts to preserve temporal continuity. For each year, the human-relevant share was calculated as the proportion of human-relevant publications relative to total OA publications. Temporal change in this proportion was evaluated using linear regression of yearly proportion on calendar year. As a sensitivity assessment, logit-transformed proportions with a continuity correction were modelled to accommodate years with low counts. Model outputs are reported in the Supplementary Materials.

### Translational stage composition analysis

To characterize the distribution of osteoarthritis (OA) research activity across major translational stages, we conducted a year-wise comparative analysis of preclinical and clinical research outputs from 1999 to 2023. Annual counts of preclinical translational activity were derived from OA-focused in vivo experimental publications, while clinical translational activity was represented by registered interventional clinical trials. These two domains were treated as distinct translational streams and were not assumed to be directly comparable in magnitude or productivity.

For each year, preclinical and clinical counts were harmonized to a common temporal window (1999–2023), with missing years explicitly set to zero to preserve continuity. A merged year-by-stage dataset was constructed, containing one observation per year per translational stage.

Year-wise absolute activity counts were summarized to represent total translational output. To assess relative distribution, within-year proportions were calculated by dividing stage-specific counts by the total activity for that year. The proportion of clinical activity was used as a summary indicator of translational stage composition over time.

Results were visualized using stacked bar charts to display absolute activity volumes, overlaid with a line plot representing the annual proportion of clinical activity. This dual representation allowed simultaneous assessment of growth in overall research activity and changes in translational stage balance without assuming proportional scaling between stages.

An exploratory binomial trend model was optionally fitted to evaluate temporal changes in clinical research share; model outputs are provided for descriptive purposes only and are reported in the Supplementary Materials.

### Assessment of experimental model reliance and human relevance in osteoarthritis research

A bibliometric analysis was conducted to evaluate the reliance of osteoarthritis (OA) research on animal-based versus human-relevant experimental models over time. Three independent, year-wise datasets covering the period from 1999 to 2023 were curated: (i) animal-based in vivo preclinical OA publications, (ii) OA publications employing human-relevant experimental models, and (iii) total OA publication counts across all study types.

Publications were classified into animal-based or human-relevant categories based on the primary experimental system emphasized in each study. These categories were defined operationally for bibliometric analysis and were not intended to represent mutually exclusive biological classifications. Studies not captured by either category (e.g., reviews, epidemiological studies, clinical observations, computational analyses, or other experimental approaches) were retained implicitly within the total OA publication counts.

Year-wise counts for animal-based and human-relevant publications were standardized and merged into a long-format dataset. Missing years within the study window were explicitly retained and assigned zero counts to ensure continuity across time. Proportions were calculated at two levels: (i) within the captured experimental set (animal-based versus human-relevant) and (ii) relative to the total OA literature.

To assess temporal trends in the relative contribution of human-relevant models within the experimental literature, a binomial generalized linear model was fitted using a logit link function, with year as a continuous predictor and the number of human-relevant publications modeled relative to animal-based publications. Model estimates, standard errors, confidence intervals, and p-values were reported.

To contextualize experimental model use within the broader OA research landscape, publication coverage was visualized by overlaying animal-based and human-relevant publication counts against the total OA publication volume for each year.

### Integrated burden–response dynamics analysis

To examine how osteoarthritis (OA) research activity evolved in relation to global disease burden between 1999 and 2023, we conducted a multi-component burden–response dynamics analysis using annual global OA disability-adjusted life years (DALYs) and publication-based research indicators. First, to assess overall scaling between burden and research output, both DALYs and annual OA publication counts were indexed to a common baseline year (1999 = 100) to enable direct comparison of relative growth trajectories. Research intensity was additionally calculated as publications per million DALYs. Proportional scaling was formally evaluated using a log–log regression model with annual publication counts as the dependent variable and DALYs as the independent variable; in this framework, the regression coefficient represents elasticity, indicating whether research output increased proportionally (β ≈ 1), less than proportionally (β < 1), or more than proportionally (β > 1) relative to disease burden.

Second, to evaluate structural redistribution within the OA research landscape, annual publication counts were categorized into three predefined domains preclinical (in vivo), human-relevant, and interventional clinical trials, and expressed as proportions of total OA publications for each year. To examine whether the internal composition of OA research shifted over time, the study period was segmented into three predefined epochs (1999–2009, 2010–2016, and 2017–2023). Within each domain and epoch, ordinary least squares regression models (proportion ~ year) were fitted to estimate absolute annual changes in domain share, expressed as percentage points per year, with 95% confidence intervals and corresponding p-values. This analysis focused on structural redistribution rather than absolute publication growth.

Third, to explore the relative contribution of research activity and disease burden to observed alignment patterns, a descriptive counterfactual decomposition was performed. Annual research output and burden were normalized as shares of their respective totals over the full observation window. An illustrative alignment distance was defined as the absolute difference between research share and burden share for each year. Using 1999 as the baseline, two counterfactual trajectories were constructed by holding either research share or burden share constant at its baseline value while allowing the other component to vary. These contrasts were descriptive and non-causal, designed to assess the sensitivity of alignment dynamics to changes in research output versus burden over time.

Finally, temporal responsiveness was examined using lagged response analysis. Year-wise DALYs were treated as the burden indicator, and three research indicators were analyzed: total OA publications, human-relevant OA publications, and interventional clinical trial counts. For each indicator, Pearson correlation coefficients were computed between burden at year *t* and research activity at year *t + lag*, with lags ranging from 0 to 10 years; only overlapping year pairs were retained for each lag. As a sensitivity assessment, simple linear regression models were fitted with research activity as the dependent variable and burden as the independent variable at corresponding lags. These analyses were exploratory and descriptive, intended to characterize lag-associated temporal alignment rather than infer causality.

### Funding acknowledgement coverage analysis

Funding coverage in osteoarthritis (OA) research was evaluated using PubMed funding metadata linked to the OA publication dataset (1999–2023). After identifying OA-related publications and extracting their PMIDs, funding acknowledgement information associated with those PMIDs was retrieved from PubMed, including grant agency name, country (when available), and grant identifiers. The funding dataset was structured in long format, where each row represented a single funding acknowledgement event linked to a publication. Publications without captured funding metadata do not appear in this dataset.

For each publication year, we calculated the number of unique OA papers with at least one captured funding acknowledgement, deduplicated by PMID. We also quantified the total number of funding acknowledgement events per year. Agency-level analyses were performed by counting the number of unique OA publications acknowledging each funding agency per year. Because publications may acknowledge multiple funders, a single publication could contribute to more than one agency category. All results reflect funding acknowledgements captured in PubMed metadata and do not represent total funding amounts, funding intensity, or the true prevalence of funded research.

### Data processing and reproducibility

All data extraction, aggregation, and classification procedures were implemented using scripted workflows in R (version 4.4.0). Publication retrieval, categorization, dataset merging, and yearly aggregation were performed programmatically using predefined query logic and rule-based classification dictionaries applied to PubMed metadata fields. No record-level manual inclusion or exclusion was conducted beyond verification of query syntax and inspection of aggregated outputs.

Classification of publications into experimental domains (e.g., human-relevant or animal-based) was determined by deterministic query definitions specified a priori. Because categorization was algorithm-driven rather than manually adjudicated, inter-rater variability does not apply. Data cleaning steps, including retention of zero-count years for temporal continuity and harmonization of year-wise datasets, were executed entirely through reproducible scripts. All statistical analyses and visualizations were conducted using R and the tidyverse ecosystem. Query definitions and classification dictionaries are described in the Supporting Information files.

### Sensitivity analysis

To evaluate robustness of research–burden alignment findings, several prespecified sensitivity analyses were conducted.

First, alignment ratios (publications per million burden) were recalculated using alternative burden denominators, including Years Lived with Disability (YLDs) and prevalence, in addition to the primary DALY-based specification. All other parameters (publication counts, normalization procedures, indexing baseline year) were held constant.

Second, to assess potential endpoint effects, indexed alignment trajectories were recalculated excluding the final year (2023), and slope comparisons were visually and descriptively examined.

Third, lag-association robustness was evaluated using YLD-based burden measures in place of DALYs for Pearson correlation analyses across 0–10 year offsets.

All sensitivity analyses were descriptive and intended to assess stability of observed temporal patterns rather than to establish causal relationships.

### Ethics statement

Ethical approval was not required for this study as it did not involve human participants, animal subjects, or the use of sensitive personal data.

## Result

### Trends in osteoarthritis research volume

**Fig 1:**
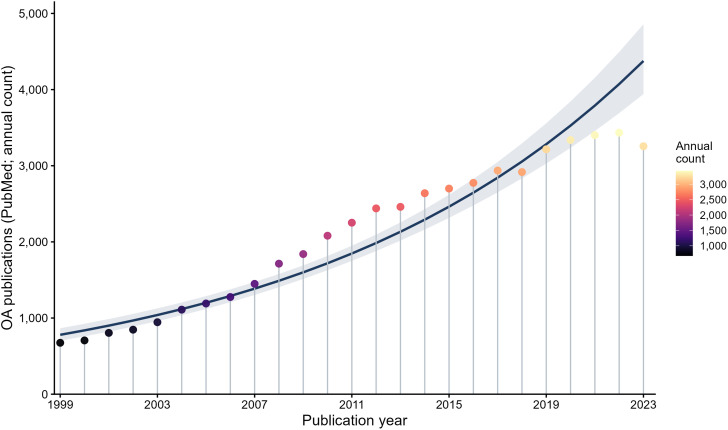
Temporal trends in osteoarthritis research volume (1999–2023). Annual counts of osteoarthritis-related publications indexed in PubMed are shown as lollipop markers, with point color intensity reflecting publication volume for each year. The solid line represents the fitted log-linear trend, and the shaded band denotes the corresponding 95% confidence interval.

Between 1999 and 2023, the annual volume of osteoarthritis (OA)-related publications indexed in PubMed increased substantially, indicating sustained growth in research activity over the study period (Fig 1). Annual publication counts rose from fewer than 1,000 publications per year in the late 1990s to over 3,000 publications per year in recent years, with particularly marked expansion beginning in the mid-2000s.

Linear regression analysis demonstrated a statistically significant positive association between calendar year and annual OA research output (S2 Table), reflecting a consistent increase in absolute publication counts over time. Log-linear modeling indicated steady proportional growth across the 25-year period. Although growth appeared to moderate slightly in the most recent years, the overall trajectory remained strongly upward.

The data demonstrate sustained long-term growth in OA-related publication volume from 1999 to 2023, providing quantitative context for subsequent analyses of research composition and temporal alignment with disease burden.

### OA disease burden

**Fig 2:**
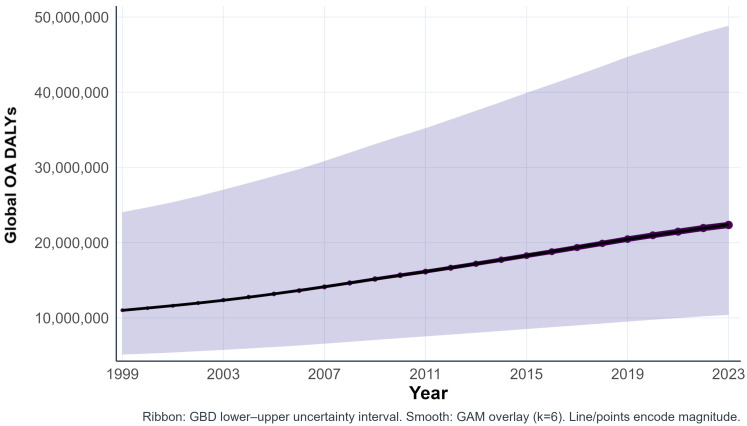
DALYs Global OA burden (Subtype-aggregated), 1999–2023. Annual global OA DALYs are shown as points connected by a line. Shaded ribbon denotes the GBD lower–upper uncertainty interval. A GAM smooth is overlaid to visually summarize the overall temporal trajectory. Values are subtype-aggregated at the global level.

Across 1999–2023, global OA disability-adjusted life years (DALYs) increased from approximately 11.0 million in 1999 to 22.36 million in 2023, representing an approximate doubling in disability burden over time (Fig 2 and S3 Table). Although uncertainty intervals were substantial in absolute magnitude, the upward trajectory was consistent across years.

Global OA prevalence increased from approximately 343.1 million in 1999 to 698.4 million in 2023, reflecting a substantial rise in the number of individuals living with OA (S1 Fig). The trajectory was consistently upward across years, with widening uncertainty intervals corresponding to the larger absolute scale of prevalence estimates.

Years lived with disability (YLDs) closely paralleled the DALY trajectory (S2 Fig), consistent with OA being predominantly a non-fatal, disability-driven condition at the global level.

Linear and log-linear models indicated statistically significant positive time trends for DALYs, YLDs, and prevalence over 1999–2023 (S4 Table). Log-linear models yielded an average annual percent increase of approximately 3% per year for each metric.

### Interventional clinical trial activity

**Fig 3:**
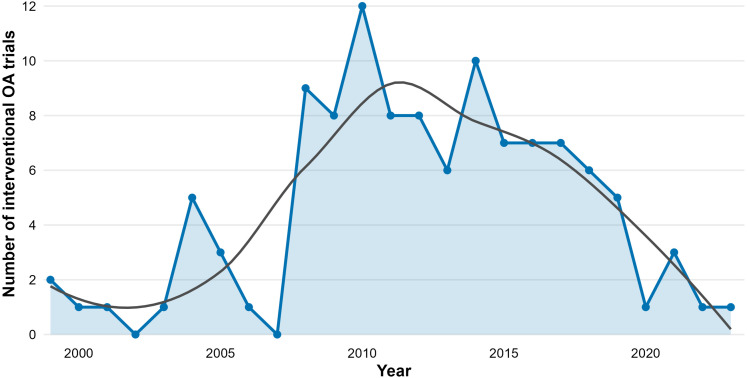
Clinical trial activity over time (Osteoarthritis). Annual counts of interventional osteoarthritis clinical trials from ClinicalTrials.gov, summarized by study start year (1999–2023). Points and line indicate yearly trial counts; shaded area provides visual emphasis of magnitude. The grey curve is a LOESS smoother included for descriptive visualization of the overall temporal pattern.

Across 1999–2023, 113 interventional OA clinical trials were identified in the registry-derived dataset (S5 Table). Trial activity was low in the early period (generally 0–2 trials per year between 1999 and 2003), increased through the mid-2000s, and reached a peak in 2010 (12 trials). Elevated activity was observed during approximately 2008–2016 (commonly 7–10 trials per year, including 10 trials in 2014) (Fig 3).

After the mid-2010s, annual trial counts declined, reaching 5 trials in 2019 and 1–3 trials per year during 2020–2023. The LOESS-smoothed curve summarizes this pattern as an initial rise followed by a gradual downturn. Lower counts in recent years should be interpreted cautiously, as registry timing and reporting completeness may influence apparent start-year distributions.

### Translational stage composition analysis

**Fig 4:**
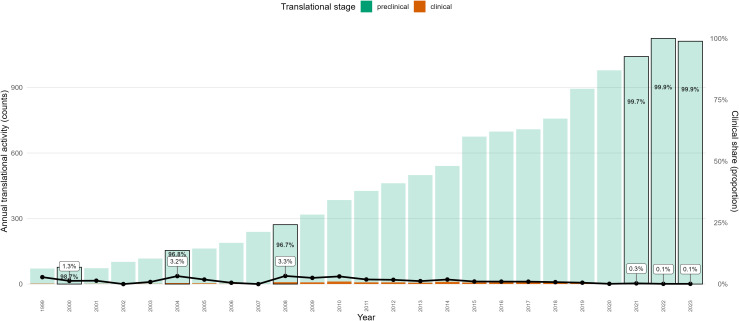
Translational stage composition in osteoarthritis research (1999–2023). Stacked bars represent annual counts of preclinical in vivo publications and registered interventional clinical trials. The overlaid line and points indicate the proportion of clinical activity relative to total translational output for each year (right y-axis). Percent labels denote within-year stage composition. This visualization jointly displays absolute research volume and relative translational stage balance over time.

Fig 4 illustrates the temporal distribution of osteoarthritis (OA) research activity across preclinical (in vivo) and interventional clinical domains between 1999 and 2023. Absolute activity increased substantially over time, primarily attributable to growth in preclinical in vivo publication counts (S6 Table).

Across the study period, preclinical research consistently represented the dominant share of captured translational activity. Although the absolute number of clinical interventional trials increased during certain intervals, their proportional contribution remained small relative to preclinical output. Exploratory trend model outputs are provided in S7 Table.

In most years, the clinical share constituted only a small percentage of the combined preclinical–clinical activity. Periods of peak overall activity continued to exhibit strong preclinical predominance, indicating that expansion in total OA research output was largely driven by upstream experimental publications rather than increases in interventional clinical trials.

### Human-relevant alignment trend in osteoarthritis research

Between 1999 and 2023, human-relevant or replacement-oriented OA research accounted for a relatively small proportion of total OA publications (mean 2.97%; range 2.03–4.25%). During this period, overall OA publication volume increased from 675 publications in 1999–3,256 publications in 2023 (approximately 4.8-fold growth). The absolute number of human-relevant publications also rose (from 17 in 1999–66 in 2023, with a peak of 114 in 2021).

**Fig 5:**
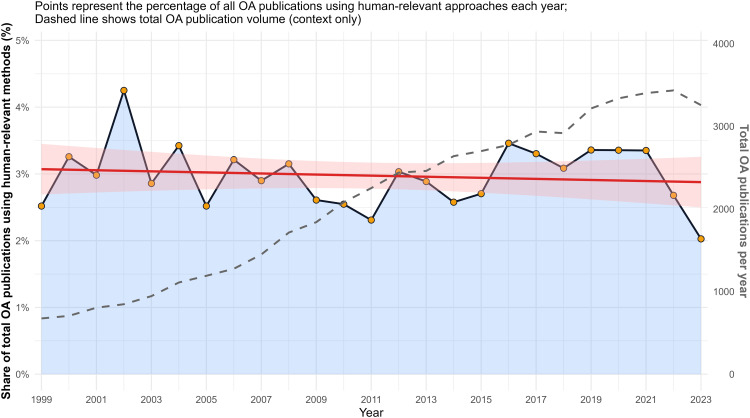
Human-relevant osteoarthritis research in the context of overall OA publication growth (1999–2023). Points show the yearly percentage of all OA publications classified as human-relevant/ 3R-aligned (replacement-oriented proxy), computed as human-relevant publications divided by total OA publications for each year. The solid line connects observed yearly proportions, and the red line shows the fitted linear trend with 95% confidence interval. The dashed line (right axis) shows total OA publication volume per year and is presented for contextual reference to illustrate overall field growth.

Proportion trend modeling did not detect a statistically significant long-term change in the share of OA research employing human-relevant methodologies (linear slope ≈ −0.008 percentage points per year, p = 0.55). These findings suggest that increases in human-relevant output largely paralleled the overall expansion of the OA literature rather than reflecting a sustained compositional shift toward replacement-oriented approaches (Fig 5).

### Temporal trends in experimental model use

**Fig 6:**
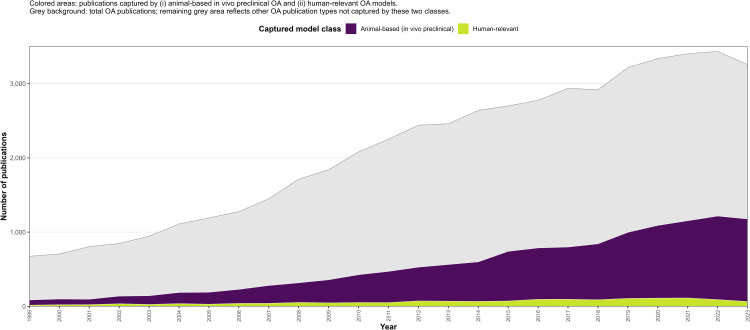
Osteoarthritis experimental model coverage within total OA literature (1999–2023). Annual publication coverage of animal-based in vivo preclinical and human-relevant experimental models within the total osteoarthritis (OA) literature from 1999 to 2023. Colored areas represent publications classified by primary experimental system as animal-based in vivo preclinical or human-relevant models. The grey background denotes the total number of OA publications in each year; the remaining grey area reflects OA publication types not captured by these two experimental categories. Classification was based on the primary experimental.

Across the study period, animal-based in vivo studies constituted the predominant experimental model within OA research. Although the absolute number of human-relevant studies increased over time, their proportional representation remained smaller relative to both animal-based experimental studies and total OA publications (Fig 6).

Within the captured experimental subset, proportion-based trend analysis indicated modest temporal variation in the relative contribution of human-relevant models. Nevertheless, animal-based approaches remained the dominant experimental modality throughout the 1999–2023 window. When considered relative to total OA publication volume, experimental model-based studies (animal and human-relevant combined) represented only a subset of overall OA research activity. These findings describe the persistent predominance of animal-based experimental models within OA research, alongside limited compositional expansion of human-relevant approaches.

## Burden–response dynamics across the OA research ecosystem

### Research–burden alignment

**Fig 7:**
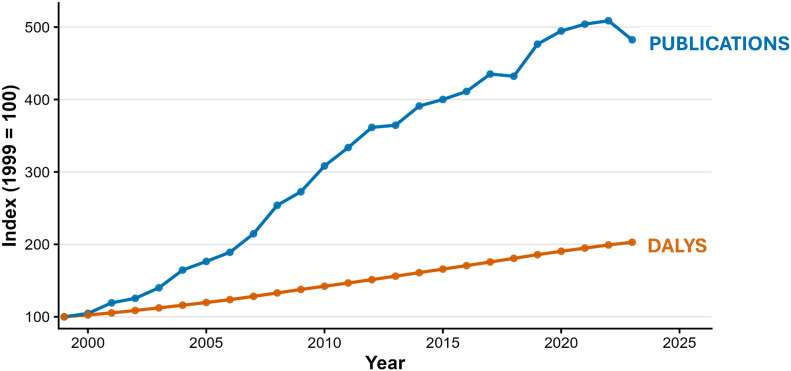
Indexed trends of osteoarthritis burden and research output (1999 = 100). Annual global osteoarthritis (OA) disability-adjusted life years (DALYs) and OA publication counts were normalized to a baseline index of 100 in 1999 to enable single-axis comparison of relative growth trajectories. The figure provides a descriptive visualization of research–burden alignment over time. DALYs increased steadily across the study period, while publication growth accelerated more rapidly, particularly after the mid-2000s.

Indexed trend analysis (1999 baseline = 100) demonstrated increasing separation in the relative growth trajectories of OA disease burden and research output over the study period (Fig 7). While global OA DALYs approximately doubled relative to baseline, OA publication counts increased by more than fivefold. The difference between indexed trajectories became more pronounced after the mid-2000s.

Consistent with this pattern, the publication-to-burden ratio (publications per million DALYs) increased steadily across 1999–2023 (S8 Table), indicating rising research intensity relative to disease burden. Log–log regression analysis yielded an elasticity coefficient greater than 1 (S9 Table), indicating that publication growth exceeded the proportional rate of DALY increase. These findings describe differential scaling behavior between burden and aggregate research output over time.

### Temporal composition change

**Fig 8:**
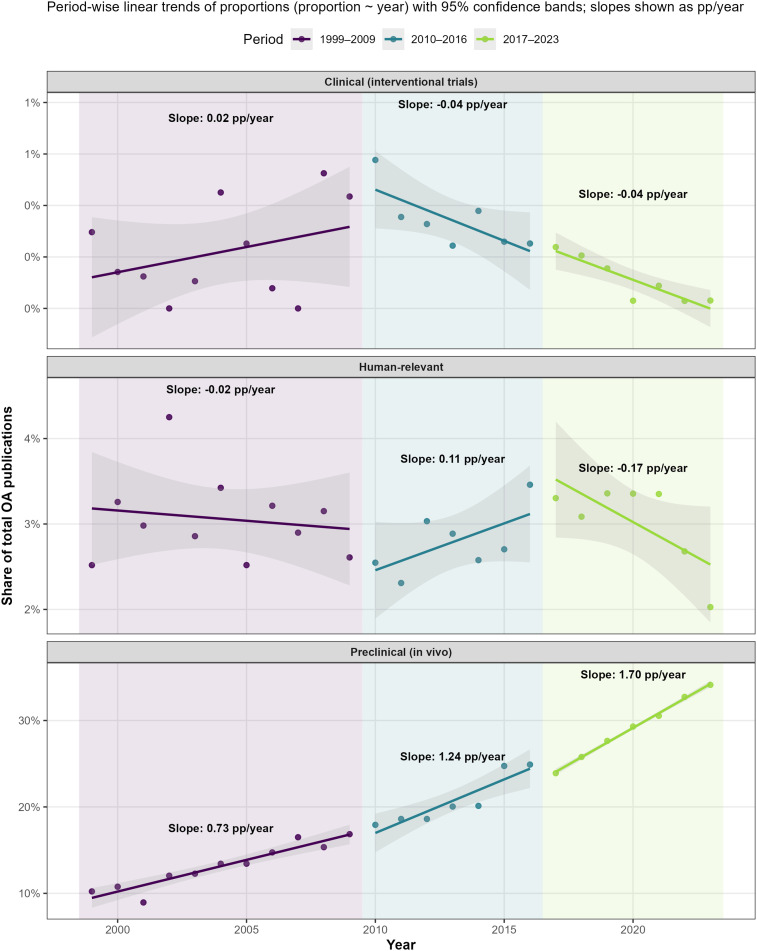
Structural (compositional) change in osteoarthritis research (1999–2023). Year-wise proportional shares of preclinical (in vivo), human-relevant, and interventional clinical trial publications relative to total OA output. The study period was divided into three predefined epochs (1999–2009, 2010–2016, and 2017–2023). Within each epoch, linear regression models (proportion ~ year) were fitted to estimate annual compositional change. Shaded regions denote 95% confidence intervals. Slopes are reported as percentage points per year (pp/year), representing absolute annual change in share.

Although total OA publication volume expanded substantially, proportional distribution across research domains evolved unevenly (Fig 8; S10 Table). Preclinical (in vivo) research demonstrated progressively increasing proportional representation across successive epochs, with estimated changes of +0.73 percentage points (pp)/year in 1999–2009, + 1.24 pp/year in 2010–2016, and +1.70 pp/year in 2017–2023.

In contrast, human-relevant research maintained a comparatively modest share. After minimal change in the earliest epoch (−0.02 pp/year), a temporary increase occurred during 2010–2016 (+0.11 pp/year), followed by decline in 2017–2023 (−0.17 pp/year). Interventional clinical trials consistently represented a small fraction of total OA publications, with marginal early growth (+0.02 pp/year) followed by modest declines (−0.04 pp/year in later epochs).

These results indicate that the expansion of OA research output was accompanied by differential changes in proportional domain representation, with increasing concentration in preclinical in vivo studies.

### Responsiveness decomposition

**Fig 9:**
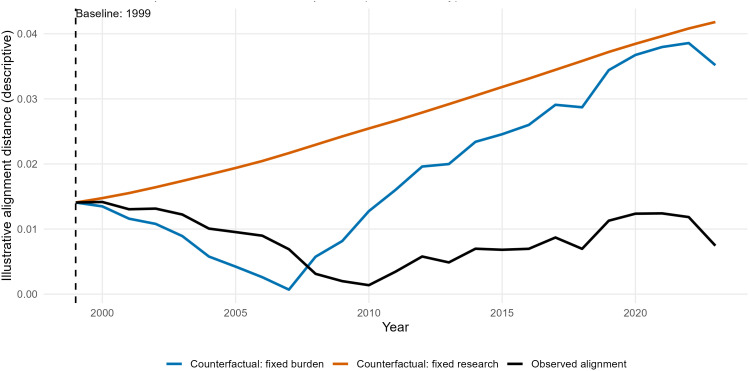
Descriptive counterfactual decomposition of OA research–burden alignment (1999–2023). Research (OA publications) and burden (OA DALYs; Global, both sexes, all ages) were normalized to shares across the full observation window, and an illustrative alignment distance was computed as |research share − burden share|. The observed trajectory (black) is contrasted with two illustrative, non-causal counterfactuals: fixed burden at baseline year 1999 (blue; burden share held constant, research share varies) and fixed research at baseline year 1999 (orange; research share held constant, burden share varies). The dashed vertical line denotes the baseline year used for counterfactual anchoring. This comparison is descriptive only and does not imply causal effects.

Descriptive counterfactual decomposition further characterized alignment patterns between research output and burden. When research output and burden were normalized as shares across the full study window, the absolute difference between research share and burden share varied modestly over time, reaching a relative minimum around 2010 before increasing slightly thereafter (Fig 9).

Holding research share constant at baseline while allowing burden to vary generated progressively larger divergence over time, whereas holding burden constant while allowing research share to vary produced early convergence followed by renewed separation. These illustrative contrasts suggest that observed alignment patterns were more sensitive to changes in research output than to changes in burden alone. All analyses are descriptive and do not imply causal responsiveness.

### Lagged response patterns

Lag-association analysis (0–10 year offsets) demonstrated consistently strong positive correlations between OA DALYs and total publication output across multiple lag intervals, indicating sustained co-trending between aggregate burden and publication growth (S3 Fig).

Human-relevant publications showed similarly positive associations at shorter lags, with attenuation at longer delays. In contrast, interventional clinical trial activity exhibited weaker contemporaneous correlations and progressively lower associations at longer lags. Given the shared upward secular trends in both burden and publication counts, these correlations primarily reflect temporal co-trending rather than independent variation.

These results describe heterogeneous temporal alignment patterns across research domains, with strong co-trending at the level of aggregate publications and more limited concordance within downstream clinical trial activity.

### Funding acknowledgement coverage in OA research (1999–2023)

The number of OA publications containing at least one funding acknowledgement in PubMed metadata increased from 53 in 1999 to a peak of 587 in 2021, followed by 527 funded papers in 2023 (S4 Fig). This represents more than a tenfold increase in metadata-captured funding acknowledgements over the study period.

The total number of funding acknowledgement events also increased over time, reflecting growth in multi-source funding patterns. These counts represent metadata-derived acknowledgements rather than direct measures of funding amounts or investment magnitude.

Agency-level patterns indicated that NIH institutes were most frequently acknowledged in OA publications, with NIAMS NIH HHS (United States) among the most commonly cited agencies. Other frequently acknowledged funders included NIA NIH HHS (United States), the Medical Research Council (United Kingdom), and CIHR (Canada) (S5 Fig).

These findings describe evolving funding acknowledgement coverage within OA research publications and should be interpreted as metadata-based patterns rather than financial investment estimates.

### Sensitivity analysis

Alignment trajectories were highly consistent when alternative burden denominators were used. Recalculation using YLDs and prevalence yielded indexed patterns similar to those observed under DALY-based alignment (S6A–S6C Fig). Exclusion of 2023 did not materially alter overall trend direction or magnitude (S6D Fig). Lag-correlation patterns using YLD-based burden measures demonstrated similar attenuation profiles across increasing offsets (S11 Table). These findings indicate that the primary alignment patterns were robust to denominator specification and endpoint variation.

## Discussion

This study provides a 25-year, system-level description of how osteoarthritis (OA) research activity has evolved relative to global disease burden. Across 1999–2023, OA disability burden approximately doubled, while aggregate publication output increased more than fivefold. Interventional clinical trial activity demonstrated a mid-period peak followed by attenuation in recent years. Human-relevant experimental approaches increased in absolute number but did not demonstrate a sustained proportional transition within the broader OA literature. These describe heterogeneous scaling patterns across translational domains rather than uniform expansion across the research-to-translation continuum.

Indexed analyses indicate that OA publication growth exceeded the proportional increase observed in global DALYs. Importantly, burden anchoring was used as a contextual reference rather than a normative expectation that research activity should scale linearly with disease burden. Biomedical publication growth has accelerated globally over the past two decades, driven by expanded funding ecosystems, technological innovation, and publication inflation dynamics [12]. Therefore, divergence between burden and publication trajectories may reflect broader structural expansion of scientific output rather than OA-specific prioritization alone.

Although interventional OA trials increased during the late 2000s and early 2010s, recent attenuation is consistent with recognized translational challenges in OA drug development. OA is increasingly understood as a heterogeneous, multi-endotype disease involving mechanical, inflammatory, metabolic, and senescence-related pathways. This biological heterogeneity complicates endpoint selection and patient stratification.

Moreover, validated surrogate biomarkers capable of predicting structural modification remain limited [13,14]. Regulatory agencies have emphasized the need for robust structural and symptomatic co-primary endpoints in DMOAD trials, increasing evidentiary thresholds for approval [13,14]. Such constraints may contribute to slower downstream clinical translation independent of overall research growth.

Thus, weaker temporal concordance between burden and trial activity may reflect structural and methodological barriers rather than a lack of research engagement.

Animal-based in vivo models remained predominant throughout the observation window. Although human-relevant systems, including organ-on-chip and microphysiological platforms have expanded across biomedical research, their integration into musculoskeletal disease modeling remains emergent [9]. Recent policy and scientific discussions emphasize that microphysiological systems require further standardization, inter-laboratory validation, and regulatory qualification before widespread adoption in drug development pipelines (https://doi.org/10.1038/s41587-020-0663-9). The modest proportional shift observed in OA research suggests that while methodological diversification is occurring, displacement of established preclinical paradigms has not yet materialized at scale.

Epoch-based compositional analyses indicate progressive concentration of proportional growth within preclinical in vivo domains. Similar asymmetries between upstream knowledge generation and downstream clinical application have been observed in broader biomedical ecosystems [6]. Translational attrition is widely recognized as a multi-stage phenomenon influenced by technical feasibility, commercial viability, regulatory requirements, and risk tolerance [11].

The present findings align with this broader literature, suggesting that expansion in publication output does not inherently guarantee equivalent growth in clinical trial initiation.

Lag-based analyses demonstrated strong co-trending between OA DALYs and total publication output, whereas clinical trial activity exhibited weaker concordance. Such divergence may reflect inherent latency in biomedical translation, where preclinical discovery does not uniformly culminate in late-stage testing. Importantly, these analyses are ecological and descriptive; they do not establish causal responsiveness or delayed adaptation.

Funding acknowledgement frequency increased substantially over time, consistent with broader growth in collaborative and multi-source funding structures. However, metadata-derived acknowledgements do not represent funding magnitude or allocation proportionality. Prior analyses of research funding alignment emphasize that financial flows are shaped by political, institutional, and strategic priorities beyond disease burden alone [4].

### Limitations

First, this study relies on aggregate, secondary datasets and therefore cannot account for trial quality, funding magnitude, or mechanistic novelty. Funding analyses reflect metadata-captured acknowledgements rather than direct financial investment. Second, bibliometric classification of experimental domains is query-driven and may be subject to misclassification despite rule-based definitions. Third, registry-based clinical trial counts may be influenced by reporting lag and jurisdiction-specific practices. Fourth, the framework is descriptive and ecological; it does not permit causal inference regarding drivers of translational change. Finally, burden anchoring provides contextual alignment rather than normative performance benchmarks.

These constraints limit interpretation but do not negate the value of systematic, multi-domain temporal mapping.

### Implications and future directions

The findings suggest that while OA research volume has expanded substantially, compositional balance across translational stages has shifted unevenly. Future work may benefit from integrating patient-level stratification data, biomarker development pipelines, and regulatory milestone tracking to better characterize translational progression dynamics. In addition, longitudinal assessment of emerging human-relevant methodologies and their integration into drug development workflows may clarify whether structural evolution accelerates in subsequent decades.

Rather than diagnosing failure, this study offers a structured descriptive lens through which temporal scaling patterns can be examined transparently. Such system-level mapping may assist policymakers, funders, and researchers in contextualizing translational dynamics within the broader constraints of biological complexity and regulatory standards.

## Conclusion

Between 1999 and 2023, osteoarthritis burden and research activity expanded concurrently but at differing rates across translational domains. Publication output demonstrated sustained and super-proportional growth relative to DALYs, whereas clinical trial activity and human-relevant experimental adoption exhibited more limited proportional shifts. These findings characterize heterogeneous scaling behavior within the OA research ecosystem and provide a burden-anchored descriptive framework for evaluating long-term translational dynamics without imposing normative assumptions of proportionality.

## Supporting information

S1 File Supplementary Material. Contains detailed PubMed query strategies, regression estimates, and all supplementary tables and figures.
