# Supplementary material for: Global burden-integrated analysis of osteoarthritis research-to-translation dynamics, 1999–2023
Source: PLoS One. 2026 May 26;21(5):e0349128. doi: 10.1371/journal.pone.0349128 (PMC13210395; doi:10.1371/journal.pone.0349128)
Supplement: S1 File — Contains detailed PubMed query strategies, regression estimates, and all supplementary tables and figures. [file pone.0349128.s001.pdf]

Supplementary Materials

Global Burden-Integrated Analysis of Osteoarthritis Research-to-Translation Dynamics, 1999–2023

Riya Mukherjee[1], Chung-Ming Chang[2*]

[1]Graduate Institute of Biomedical Sciences, Chang Gung University, Taoyuan, Taiwan
[2]Master & PhD program in Biotechnology Industry, Chang Gung University

***Corresponding author**

Chung-Ming Chang*, Master & Ph.D. program in Biotechnology Industry, Chang Gung University, No.259, Wenhua 1st Rd., Guishan Dist. Taoyuan City 33302, Taiwan (R.O.C.), cmchang@mail.cgu.edu.tw (C.M.C.).

MeSH Terms used

OA Translational Ecosystem PubMed Queries (1999–2023)

NOTE: Year-specific filter appended in code for each year Y:
AND ("Y/01/01"[dp] : "Y/12/31"[dp])

1) OA total publications (denominator) — BASE QUERY:
(osteoarthritis[mh]) NOT (animals[mh] NOT humans[mh]) NOT (editorial[pt] OR comment[pt] OR letter[pt] OR news[pt] OR published erratum[pt] OR review[pt] OR systematic review[pt] OR meta-analysis[pt])

2) OA + human-relevant/Replacement subset (numerator) — BASE QUERY:
(osteoarthritis[mh]) AND (in vitro techniques[mh] OR tissue engineering[mh] OR microfluidics[mh] OR scaffolds[mh] OR hydrogels[mh] OR bioprinting[mh] OR organoids[mh] OR (organ-on-chip[tiab] OR "organ on a chip"[tiab] OR "organ-on-a-chip"[tiab] OR microphysiological[tiab] OR "microphysiological system"[tiab] OR "microphysiological systems"[tiab] OR "lab-on-a-chip"[tiab] OR "lab on a chip"[tiab] OR microfluidic*[tiab] OR scaffold*[tiab] OR hydrogel*[tiab] OR biomaterial*[tiab] OR bioprint*[tiab] OR "3d bioprint*"[tiab] OR "3d print*"[tiab] OR "3-d print*"[tiab] OR explant*[tiab] OR "cartilage explant*"[tiab] OR "tissue explant*"[tiab] OR "3d culture"[tiab] OR "3-d culture"[tiab] OR "three-dimensional culture"[tiab] OR spheroid*[tiab] OR organoid*[tiab] OR "3d scaffold*"[tiab] OR "3-d scaffold*"[tiab] OR "tissue engineered"[tiab] OR "tissue-engineered"[tiab])) NOT (animals[mh] NOT humans[mh]) NOT (editorial[pt] OR comment[pt] OR letter[pt] OR news[pt] OR published erratum[pt] OR review[pt] OR systematic review[pt] OR meta-analysis[pt])

OA PubMed Query — Preclinical In Vivo (Animal Model Validation Proxy) 1999–2023

==

NOTE: Year-specific filter appended in code for each year Y:
AND ("Y/01/01"[dp] : "Y/12/31"[dp])

BASE QUERY:

(osteoarthritis[mh]) AND (animals[mh]) AND (disease models, animal[mh] OR animal experimentation[mh] OR in vivo[tiab] OR model*[tiab]) NOT (editorial[pt] OR comment[pt] OR letter[pt] OR news[pt] OR published erratum[pt] OR review[pt] OR systematic review[pt] OR meta-analysis[pt]) NOT (case reports[pt]) NOT (clinical trial[pt] OR randomized controlled trial[pt] OR controlled clinical trial[pt] OR clinical study[pt])

Supplementary Table S1. Annual counts of PubMed-indexed osteoarthritis-related publications from 1999 to 2023.

Year	OA_publication_count
1999	675
2000	706
2001	805
2002	847
2003	945
2004	1110
2005	1191
2006	1276
2007	1449
2008	1714
2009	1840
2010	2081
2011	2252
2012	2440
2013	2460
2014	2639
2015	2700
2016	2775
2017	2937
2018	2917
2019	3216
2020	3338
2021	3402
2022	3434
2023	3256

Supplementary Table S2. Regression estimates for temporal trends in osteoarthritis research volume, including linear and log-linear model coefficients with 95% confidence intervals and p-values.

model	term	estimate	conf.low	conf.high	p.value

Linear (count ~ year)	(Intercept)	-257067.6	-272689.4	-241445.7	3.51E-21
Linear (count ~ year)	year	128.87308	121.10493	136.64122	2.93E-21
Log-linear (log(count) ~ year)	(Intercept)	-136.9983	-152.8692	-121.1274	5.63E-15
Log-linear (log(count) ~ year)	year	0.0718646	0.0639726	0.0797566	1.78E-15

Supplementary Table S3. Yearly global OA DALYs, 1999–2023.
Annual global OA DALYs with corresponding lower and upper GBD uncertainty bounds.

Year	Global_OA_DALYs	DALYs_Lower	DALYs_Upper
1999	11020681	5126885	24054387
2000	11296740	5257764	24698334
2001	11612648	5401352	25386870
2002	11980268	5573716	26181580
2003	12373563	5751882	27050467
2004	12776338	5937560	27935474
2005	13189489	6130390	28857331
2006	13626894	6339090	29779206
2007	14125530	6572340	30858035
2008	14647968	6823226	31974342
2009	15172210	7071324	33106141
2010	15672104	7314749	34182988
2011	16156767	7546981	35227331
2012	16671316	7788925	36380541
2013	17198892	8027566	37547468
2014	17735149	8273258	38715159
2015	18276455	8525631	39923443
2016	18801754	8767973	41080899
2017	19355745	9012180	42259782
2018	19910013	9255276	43463708
2019	20476977	9514658	44712721
2020	20988075	9753633	45798419
2021	21467463	9983772	46880227
2022	21950371	10221361	47939196
2023	22357934	10404685	48849358

Supplementary Materials

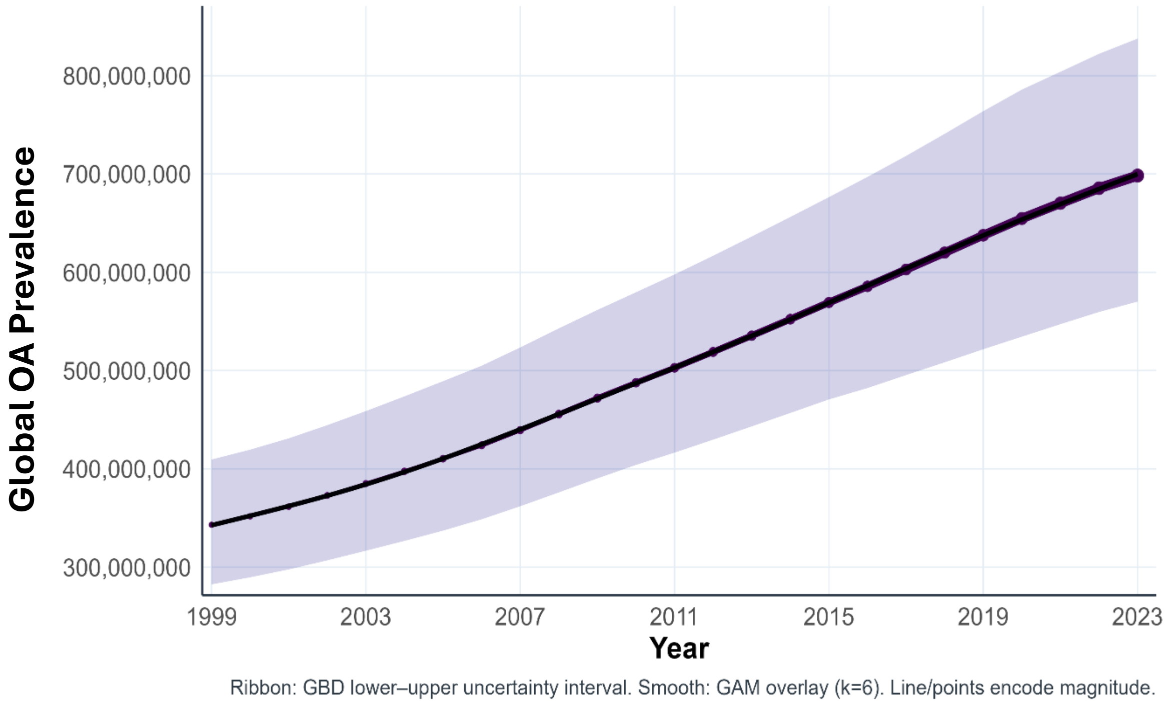

Figure S1. Prevalence- Global OA burden (Subtype-aggregated), 1999–2023. Annual global OA prevalence is shown as points connected by a line. Shaded ribbon denotes the GBD lower–upper uncertainty interval. A GAM smooth is overlaid to summarize the long-term trajectory. Values are subtype-aggregated at the global level.

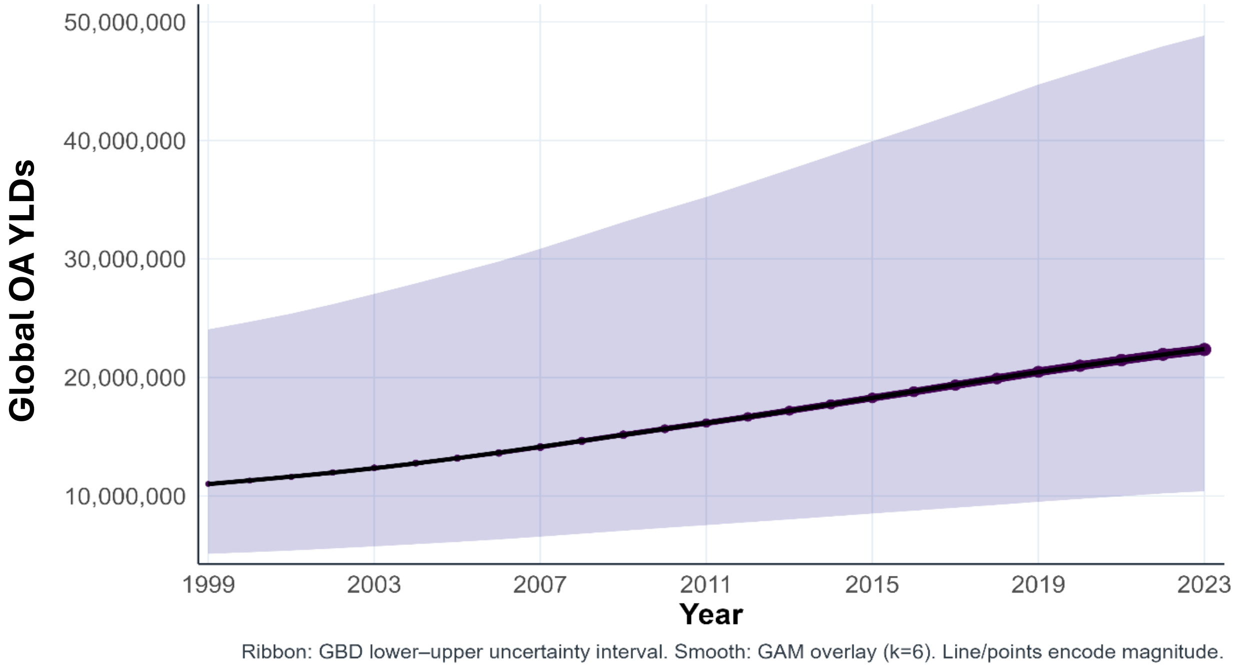

Figure S2. YLDs-Global OA burden (Subtype-aggregated), 1999–2023.
Annual global OA YLDs are shown as points connected by a line. Shaded ribbon denotes the GBD lower–upper uncertainty interval. A GAM smooth is overlaid. The YLD trajectory closely matches DALYs, consistent with disability-dominant OA burden at the global scale.

Supplementary Table S4. Regression-derived trend summaries for OA burden metrics
Linear and log-linear regression outputs for DALYs, YLDs, and prevalence, including estimated coefficients, confidence intervals, p-values, and derived average annual percent change from log-linear models.

metric	model	term	estimate	conf.low	conf.high	p.value
DALYs	Linear (burden ~ year)	(Intercept)	-9.8E+08	-1E+09	-9.5E+08	1.10E-29
DALYs	Linear (burden ~ year)	year	493390.8	480900.8	505880.9	7.52E-30
DALYs	Log-linear (log(burden) ~ year)	(Intercept)	-45.2133	-46.6031	-43.8235	6.43E-28
DALYs	Log-linear (log(burden) ~ year)	year	0.03073	0.030039	0.031422	4.99E-31
DALYs	Derived: Avg annual % change (from log-linear slope)	year	3.120752	3.049509	3.192045	4.99E-31
YLDs	Linear (burden ~ year)	(Intercept)	-9.8E+08	-1E+09	-9.5E+08	1.10E-29
YLDs	Linear (burden ~ year)	year	493390.8	480900.8	505880.9	7.52E-30
YLDs	Log-linear (log(burden) ~ year)	(Intercept)	-45.2133	-46.6031	-43.8235	6.43E-28
YLDs	Log-linear (log(burden) ~ year)	year	0.03073	0.030039	0.031422	4.99E-31
YLDs	Derived: Avg annual % change (from log-linear slope)	year	3.120752	3.049509	3.192045	4.99E-31
Prevalence	Linear (burden ~ year)	(Intercept)	-3.1E+10	-3.1E+10	-3E+10	3.12E-29
Prevalence	Linear (burden ~ year)	year	15435579	15026642	15844517	2.14E-29
Prevalence	Log-linear (log(burden) ~ year)	(Intercept)	-42.0091	-43.3198	-40.6984	9.03E-28
Prevalence	Log-linear (log(burden) ~ year)	year	0.030847	0.030195	0.031499	1.19E-31
Prevalence	Derived: Avg annual % change (from log-linear slope)	year	3.132771	3.065576	3.20001	1.19E-31

Supplementary Table S5. Annual interventional OA trial counts (1999–2023).

Year-by-year number of interventional osteoarthritis trials from ClinicalTrials.gov summarized using the study start year. Years with no trials are retained as zero counts to preserve a complete time series.

start_year	n_interventional_trials
1999	2
2000	1
2001	1
2003	1
2004	5
2005	3
2006	1
2008	9
2009	8
2010	12
2011	8
2012	8
2013	6
2014	10
2015	7
2016	7
2017	7
2018	6
2019	5
2020	1
2021	3
2022	1
2023	1

Supplementary Table S6. Annual counts and proportions of preclinical and clinical OA research activity (1999–2023).

model	term	estimate_type	estimate	conf.low	conf.high	p.value
Binomial GLM: clinical proportion ~ year	Intercept	Odds Ratio (exp(beta))	9.45E+83	3.69E+59	1.36E+108	1.13E-11
Binomial GLM: clinical proportion ~ year	Year (per 1-year increase)	Odds Ratio (exp(beta))	0.906366	0.881648	0.93203	3.77E-12

Supplementary Table S7. Temporal trend analysis of clinical research proportion in OA.

year	stage	n	total_year	proportion
1999	clinical	2	71	0.028169
1999	preclinical	69	71	0.971831

Supplementary Materials

2000	clinical	1	77	0.012987
2000	preclinical	76	77	0.987013
2001	clinical	1	73	0.013699
2001	preclinical	72	73	0.986301
2002	clinical	0	102	0
2002	preclinical	102	102	1
2003	clinical	1	117	0.008547
2003	preclinical	116	117	0.991453
2004	clinical	5	154	0.032468
2004	preclinical	149	154	0.967532
2005	clinical	3	163	0.018405
2005	preclinical	160	163	0.981595
2006	clinical	1	189	0.005291
2006	preclinical	188	189	0.994709
2007	clinical	0	239	0
2007	preclinical	239	239	1
2008	clinical	9	272	0.033088
2008	preclinical	263	272	0.966912
2009	clinical	8	318	0.025157
2009	preclinical	310	318	0.974843
2010	clinical	12	385	0.031169
2010	preclinical	373	385	0.968831
2011	clinical	8	427	0.018735
2011	preclinical	419	427	0.981265
2012	clinical	8	462	0.017316
2012	preclinical	454	462	0.982684
2013	clinical	6	499	0.012024
2013	preclinical	493	499	0.987976
2014	clinical	10	541	0.018484
2014	preclinical	531	541	0.981516
2015	clinical	7	675	0.01037
2015	preclinical	668	675	0.98963
2016	clinical	7	698	0.010029
2016	preclinical	691	698	0.989971
2017	clinical	7	709	0.009873
2017	preclinical	702	709	0.990127
2018	clinical	6	758	0.007916
2018	preclinical	752	758	0.992084
2019	clinical	5	894	0.005593
2019	preclinical	889	894	0.994407
2020	clinical	1	979	0.001021
2020	preclinical	978	979	0.998979

2021	clinical	3	1042	0.002879
2021	preclinical	1039	1042	0.997121
2022	clinical	1	1125	8.89E-04
2022	preclinical	1124	1125	0.999111
2023	clinical	1	1112	8.99E-04
2023	preclinical	1111	1112	0.999101

Supplementary Table S8. Annual OA research intensity expressed as publications per million DALYs (1999–2023).

Year	Publications	DALYs	Publications_per_million_DALYs
1999	675	11020681	61.24848
2000	706	11296740	62.4959
2001	805	11612648	69.32097
2002	847	11980268	70.69959
2003	945	12373563	76.3725
2004	1110	12776338	86.87935
2005	1191	13189489	90.29918
2006	1276	13626894	93.63836
2007	1449	14125530	102.5802
2008	1714	14647968	117.0128
2009	1840	15172210	121.2744
2010	2081	15672104	132.7837
2011	2252	16156767	139.3843
2012	2440	16671316	146.3592
2013	2460	17198892	143.0325
2014	2639	17735149	148.8006
2015	2700	18276455	147.7311
2016	2775	18801754	147.5926
2017	2937	19355745	151.7379
2018	2917	19910013	146.5092
2019	3216	20476977	157.0544
2020	3338	20988075	159.0427
2021	3402	21467463	158.4724
2022	3434	21950371	156.4438
2023	3256	22357934	145.6306

Supplementary Table S9. Log–log regression model assessing proportional scaling between OA publications and DALYs.

model	term	estimate	conf.low	conf.high	std.error	statistic	p.value

log(n_publications) ~ log(dalys)	(Intercept)	-31.62187	-35.10908	-28.13467	1.6857321	-18.75854	1.95E-15
log(n_publications) ~ log(dalys)	log(dalys)	2.3600707	2.1498355	2.5703059	0.1016288	23.22246	1.82E-17

Supplementary Table S10. Period-wise slope estimates for compositional change. Linear regression estimates of annual change in proportional share (percentage points per year) within predefined epochs (1999–2009, 2010–2016, 2017–2023) for each research domain. Slopes, 95% confidence intervals, and p-values are reported.

period	category	slope	CI_lower	CI_upper	p_value
1999-2009	Clinical (interventional trials)	1.96E-04	-1.99E-04	5.91E-04	0.290811
2010-2016	Clinical (interventional trials)	-3.98E-04	-8.16E-04	2.00E-05	0.058101
2017-2023	Clinical (interventional trials)	-3.73E-04	-5.73E-04	-1.72E-04	0.004991
1999-2009	Human-relevant	-2.40E-04	-0.00136	8.78E-04	0.63904
2010-2016	Human-relevant	0.001097	-4.74E-04	0.002668	0.132653
2017-2023	Human-relevant	-0.00166	-0.00354	2.24E-04	0.072888
1999-2009	Preclinical (in vivo)	0.007331	0.005406	0.009255	1.22E-05
2010-2016	Preclinical (in vivo)	0.012398	0.006186	0.018611	0.003677
2017-2023	Preclinical (in vivo)	0.01695	0.015916	0.017984	1.42E-07

Supplementary Materials

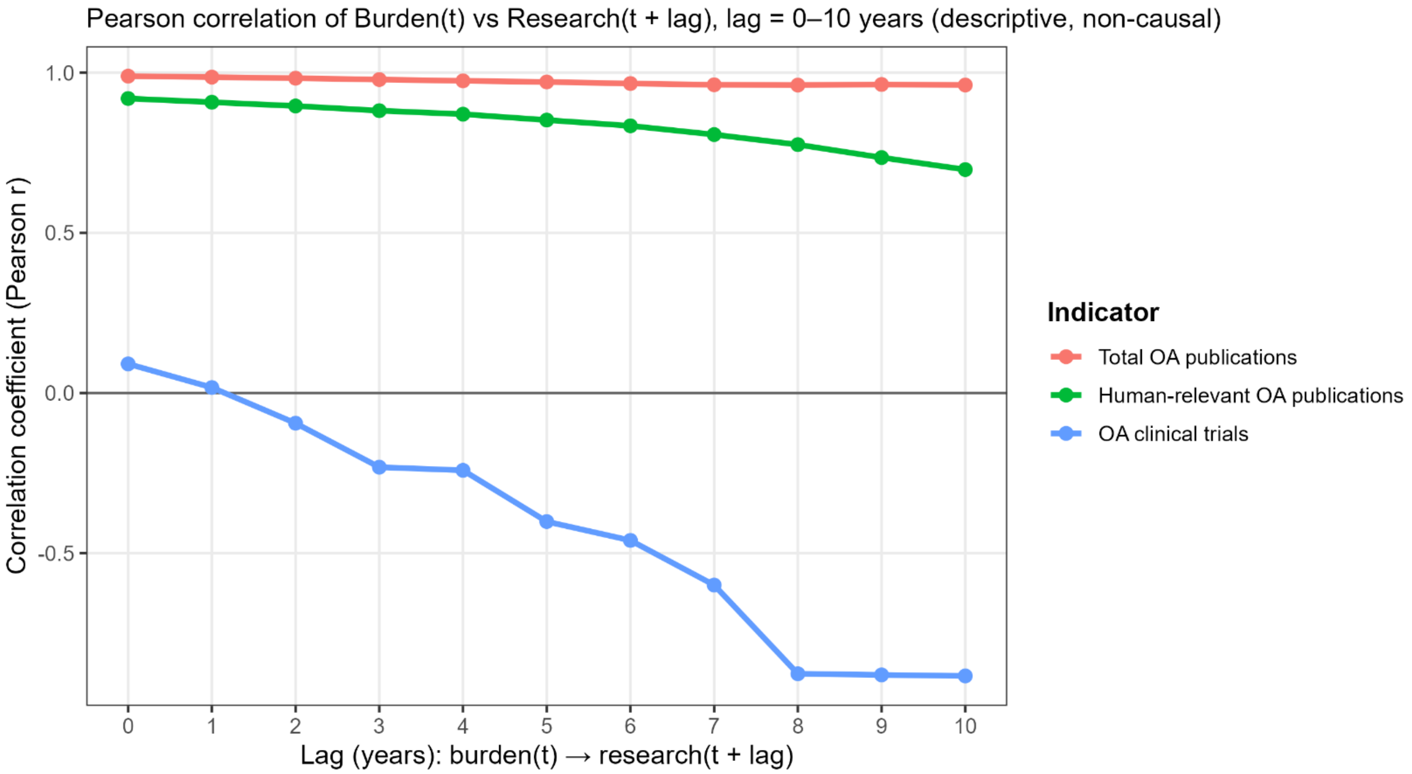

Figure S3. Lag-association between OA burden and subsequent research activity. Pearson correlation coefficients between global osteoarthritis (OA) DALYs at year *t* and research indicators at year *t* + *lag* for lags ranging from 0 to 10 years. Indicators include total OA publications, human-relevant OA publications, and OA interventional clinical trials. Positive values indicate temporal alignment between burden and later research activity, whereas negative values indicate divergence. This analysis is descriptive and does not imply causal or responsive effects.

Supplementary Materials

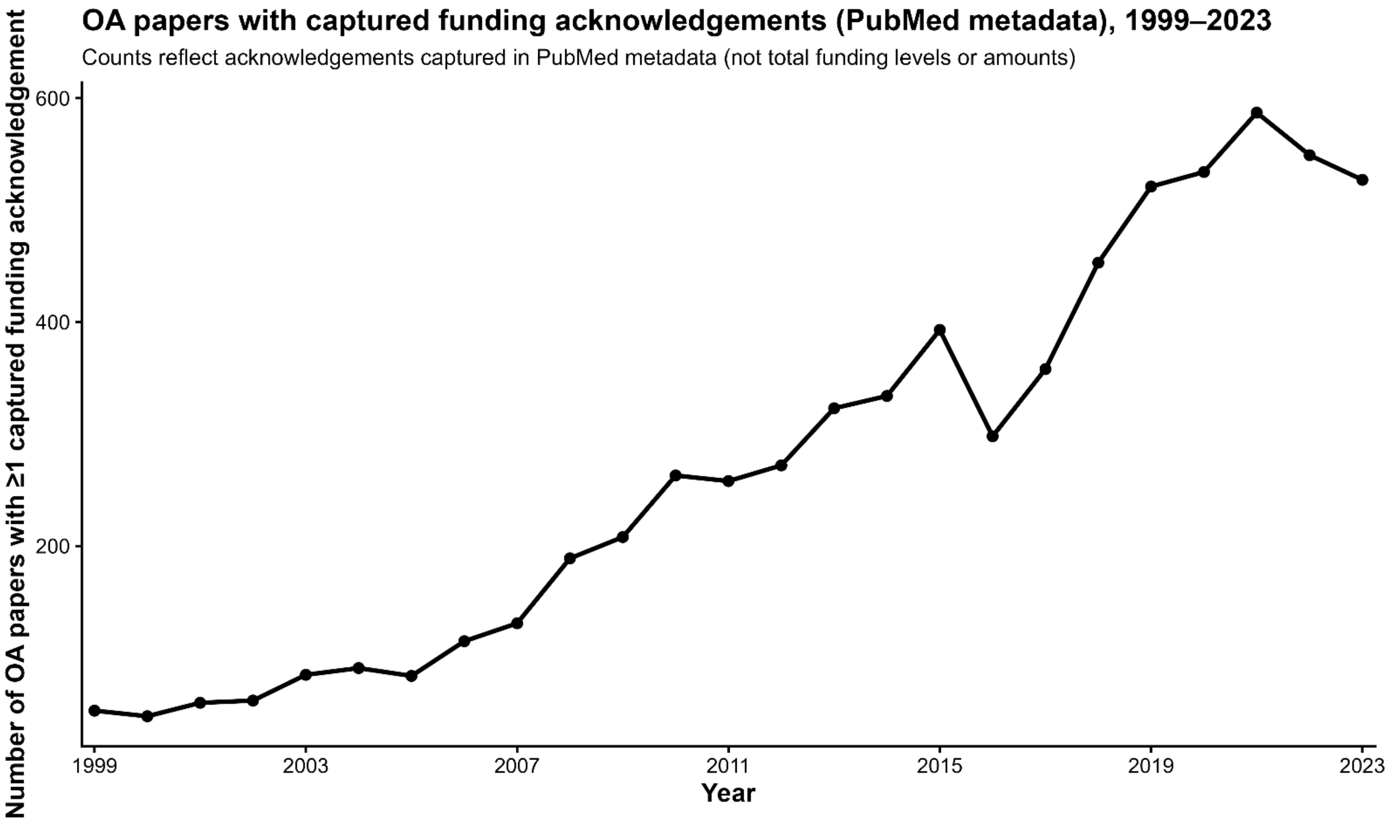

Figure S4. Funding acknowledgement presence in OA research (1999–2023). Number of OA publications with ≥1 funding acknowledgement captured in PubMed metadata. Counts reflect funding acknowledgements recorded in PubMed and do not represent total funding amounts or funding prevalence.

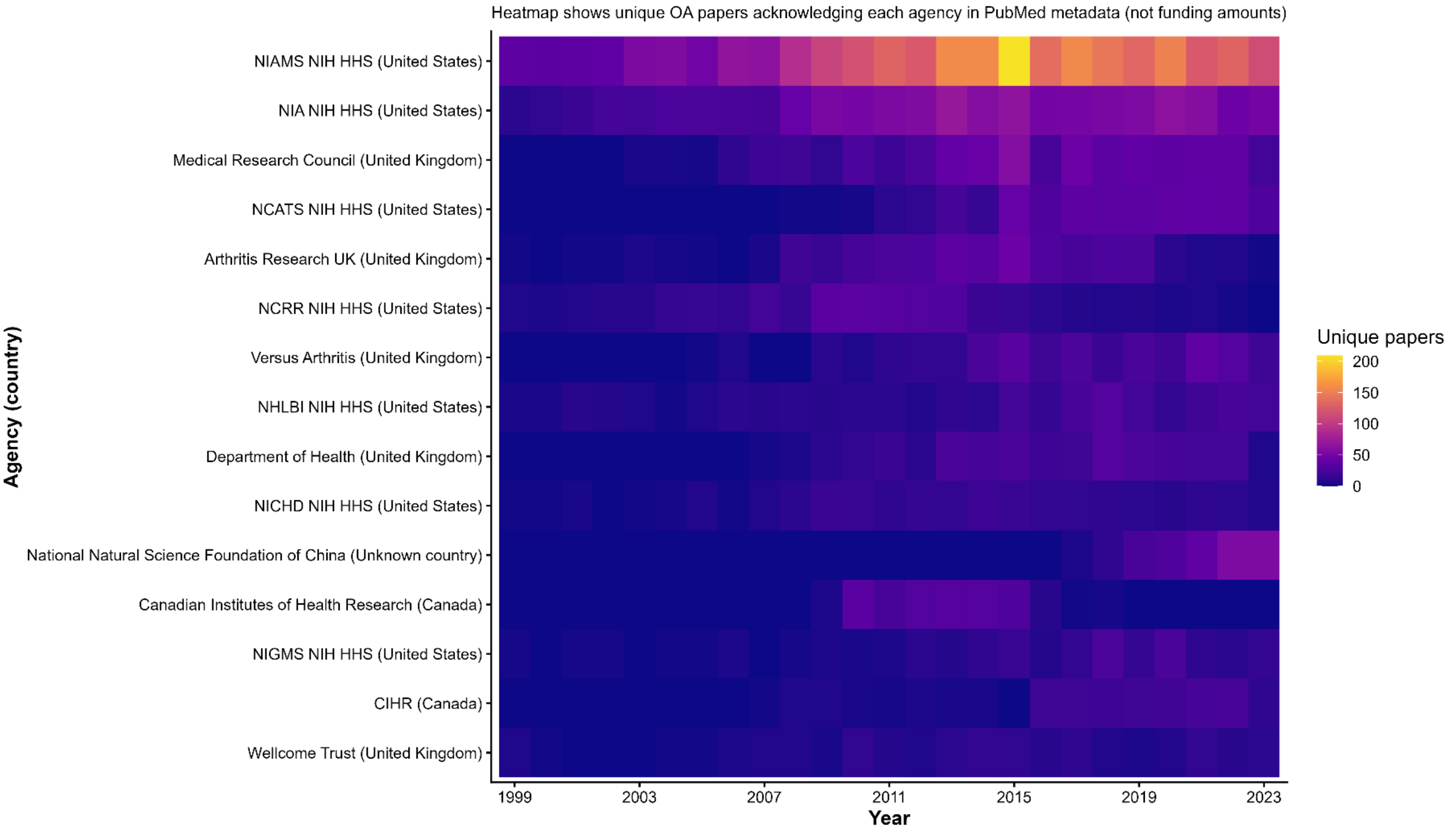

Figure S5. Top 15 funding agencies acknowledged in OA publications (1999–2023). Heatmap shows the number of unique OA publications acknowledging each funding agency per year, based on PubMed metadata. A single publication may acknowledge multiple agencies. Counts reflect metadata-captured acknowledgements and not funding amounts.

Supplementary Materials

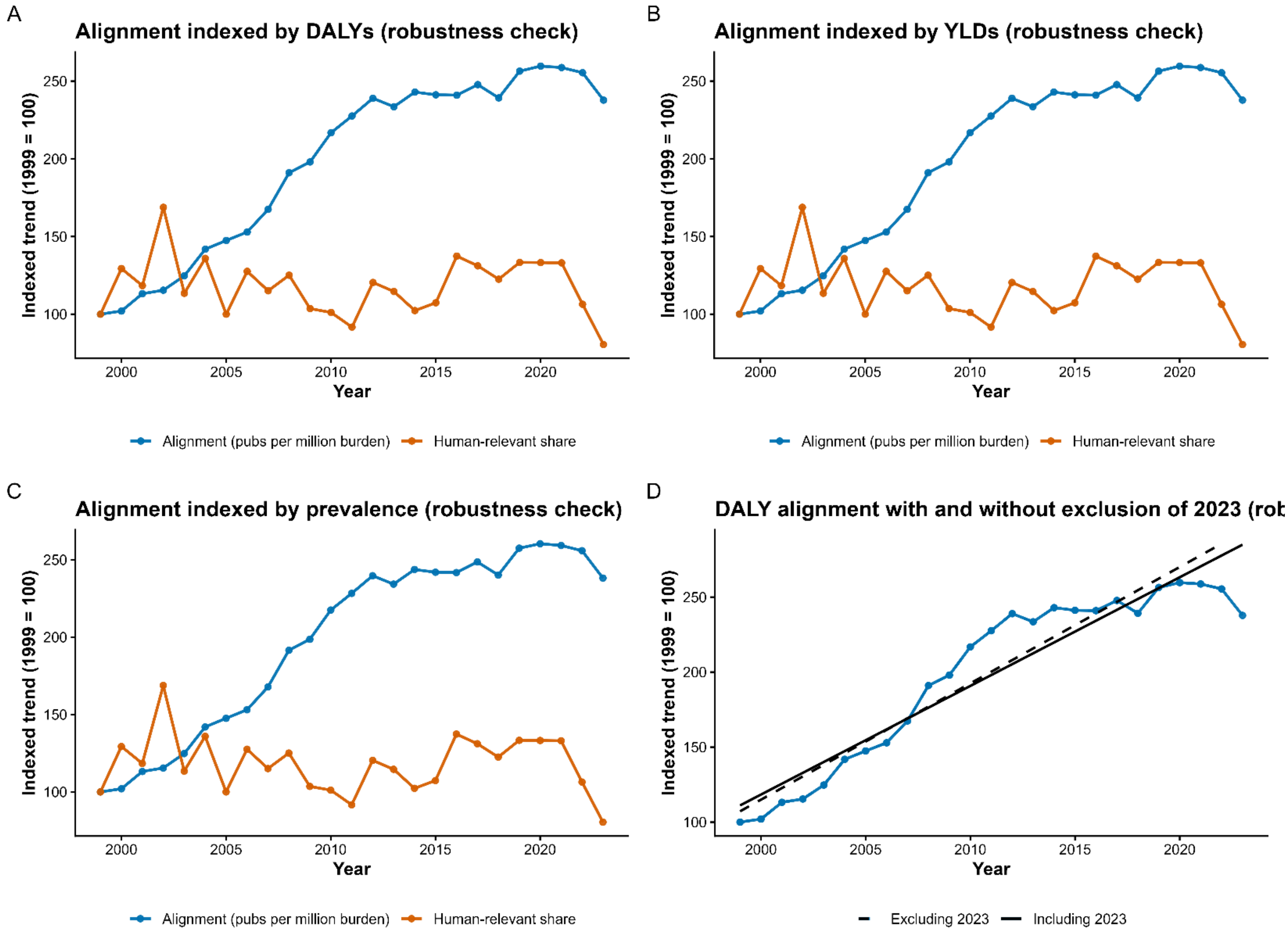

Figure S6. Sensitivity analyses of research–burden alignment (1999–2023).

(A) Alignment indexed using DALYs (primary specification).
(B) Alignment indexed using Years Lived with Disability (YLDs) as alternative burden denominator.
(C) Alignment indexed using prevalence as alternative burden denominator.
In panels A–C, indexed trends are normalized to 1999 = 100. Blue curves represent alignment (publications per million burden), and orange curves represent the share of human-relevant publications.

(D) DALY-based alignment trajectory including and excluding 2023 to evaluate endpoint stability.

Supplementary Table S11. Lag-association sensitivity analysis using YLD-based burden.

Pearson correlation coefficients between burden(t) and research(t + lag) for lags 0–10 years using YLD-based burden measures. Results are descriptive and non-causal.

lag_years	n_pairs	spearman_r
0	25	0.9946154
1	24	0.993913
2	23	0.993083
3	22	0.9920949
4	21	0.9909091
5	20	0.9894737
6	19	0.9877193
7	18	0.9855521
8	17	0.9828431
9	16	0.9794118
10	15	0.975
